# Supplementary material for: Path loss measurement and modeling of 5G network in emergency indoor stairwell at 3.7 and 28 GHz
Source: PLoS One. 2023 Mar 28;18(3):e0282781. doi: 10.1371/journal.pone.0282781 (PMC10047544; doi:10.1371/journal.pone.0282781)
Supplement: S1 Appendix — (PDF) [file pone.0282781.s005.pdf]

Appendix. See Table 8

Table 8. Meanings of the used symbols.

Symbols	Meanings
α	intercepting parameter in unit dB in ABM and ABGM model
β	slope of the line in ABM and ABGM model
γ	statistical parameters of the ABGM model
σ	standard deviation of Gaussian distribution
b	optimization specification parameter in ABGM model
$\mathrm{FSPL}(f, d_0)$	projected path loss at d_0 where f is the carrier frequency
n	path loss exponent
P_{ABGM}	path loss of ABGM model
P_{ABM}	path loss of ABM model
P_{CIDF}	path loss of CIDF model
P_{CIDMF}	path loss of CIDMF model
ξ	random variable with a zero-mean Gaussian distribution
$C_{\mathrm{loss},r}$	attenuation in the transmission cable in receiver side
$C_{\mathrm{loss},t}$	attenuation in the transmission cable in transmitter side
d, d_0	distance
G_{ar}	receiver antenna gain in decibel
G_{at}	transmitter antenna gain in decibel
P_r	received signal level at the receiver side
P_t	transmitted signal strength level
P	path loss
