# Supplementary material for: Path loss measurement and modeling of 5G network in emergency indoor stairwell at 3.7 and 28 GHz
Source: PLoS One. 2023 Mar 28;18(3):e0282781. doi: 10.1371/journal.pone.0282781 (PMC10047544; doi:10.1371/journal.pone.0282781)
Supplement: Attachment — Submitted filename: Response to Reviewers.docx [file pone.0282781.s006.docx]

**Response to Reviewers**

**Reviewer #1:** The paper covers a timely and interesting topic. This reviewer has the below comments.

1. The Author’s should provide a table summarizing the main notation that has been adopted in the manuscript.

Author’s response: We have the summarizing table containing all the symbols used in Table 8.

2. Author’s need to cover a few operational frequencies to make the work more robust and prove the accuracy of the work. If it is not possible due to measurement data then should refer to the operation frequency in the title.

Author’s response: We have used the operating frequency in the manuscript title.

3. Need to present some clear modelling layouts where signal details information should be found. For more details, the Author’s can read some recent works regarding radio propagation modelling such as (<https://doi.org/10.1016/j.aej.2022.04.033,> <https://doi.org/10.1371/journal.pone.0201905,> and <https://doi.org/10.3390/electronics8030286> ).

Author’s response: We have added Tables 1, 2, and 3 and procedure to calculate path loss from the received power in lines 159—173.
4. References are not up to date. Need to align with the literature on indoor radio propagation research.

Author’s response: We have revised the introduction and background study by adding related indoor radio propagation research work. (lines 4—22)

5. Proposed method detail is missing. Need to demonstrate the proposed method performance with respect of any other benchmark method .

Author’s response: We have already compared our results with the available results in the literature about the path measurement result in the emergency stair in Table 6 and 7.

6. Simulation details information needed including simulation configuration parameters such as Antenna gain, cable loss etc.

Author’s response: We have given the real measurement configuration parameters in this version of the manuscript (Tables 1, 2, and 3).

**Reviewer #2:** This is a very difficult paper to review. It needs to put the images embedded in the text where they occur. This is obviously the best thing to do as it is not at all easy scrolling down to an image from the text and being unsure which one it is referring to.

Author’s response: According to the format of this journal, we cannot insert images inside the text—the current style of submission of this journal during the review process.

Here an empirical propagation model has been measured for propagation through stair wells. This should be done for different locations to show consistency/inconsistency. But then is measuring the stairwell only of any relevance? Rather what is needed is to have a horn antenna illuminate the bottom of the stairwell to represent the extra loss likely to be found from any EM waves entering the stairwell from the floor. Then the antenna up the stairwell would be the mobile and thus should be omnidirectional. Really what is needed is samples of measurements up the stairs vs height, so that then it can be marked clearly where each floor is and then some idea be given as to how the propagation works and if path loss exponents do change. This is not clear from the data and certainly only data need be presented as path losses, not power received.

Author’s response: We have already analyzed the consistency/inconsistency of the measured data in table 4.

Please see other measurement scenarios in the literature, for example, (1) doi:10.3906/elk-1710-248, (2) doi:10.1109/LAWP.2019.2961641, about placing the antenna in the emergency stair.

We have added a description of how the path losses were calculated from the received power (in lines 159—173).

For mmWave this is not so useful information given it is NLOS and does not really work well at that band up stairs with line of sight blockage. With the need for directive antennas it is hard to determine the max position where there is loss or has it been averaged based on taking all azimuth directions? The elevation would likewise also matter there.

Author’s response: The literature uses different antenna configuration types in the studied wireless link. Various researchers used horn-to-horn, dipole-to-dipole, or omnidirectional-to-horn type antennae in the reported study (please see other research works as reported in Table 4.). In this study, we used the horn antenna in the transmitter and the antenna in the receiver in a special type of stair environment (stairs are separated by wall) that has not been studied before.

This paper certainly can do with more clear documentaton of results that show the real actual path loss and not power levels as well as ensuring the interpretation of them.

Author’s response: We have already simulated the actual path loss in Figures 8, 9, and 10. In addition, calculated path losses are given in Tables 1, 2, and 3.

**Reviewer #3:** The paper is good and technically sound. The measurements are designed and presented in a good way. However, two minor issues need to be revised in order to improve the paper quality
1- the abstract is not consice enough and need to be rewritten in better way to give more insights about the motivation and contributions

Author’s response: We have revised the abstract.

2- More dissuction about the motivation and research gap should be added in the introduction.

Author’s response: We have revised the motivation and research gap in lines 5—22, 38—42, and 74—81.
