# Supplementary material for: Path loss measurement and modeling of 5G network in emergency indoor stairwell at 3.7 and 28 GHz
Source: PLoS One. 2023 Mar 28;18(3):e0282781. doi: 10.1371/journal.pone.0282781 (PMC10047544; doi:10.1371/journal.pone.0282781)
Supplement: Attachment — Submitted filename: Response to Reviewers.docx [file pone.0282781.s007.docx]

**Response to Reviewers**

**Reviewer #2**

1. If the authors have adequately addressed your comments raised in a previous round of review and you feel that this manuscript is now acceptable for publication, you may indicate that here to bypass the “Comments to the Author” section, enter your conflict of interest statement in the “Confidential to Editor” section, and submit your "Accept" recommendation.

Reviewer #2: All comments have been addressed

Author’s response: We want to thank the reviewer.

2. Is the manuscript technically sound, and do the data support the conclusions?

The manuscript must describe a technically sound piece of scientific research with data that supports the conclusions. Experiments must have been conducted rigorously, with appropriate controls, replication, and sample sizes. The conclusions must be drawn appropriately based on the data presented.

Reviewer #2: Partly

Author’s response: We want to thank the reviewer.

3. Has the statistical analysis been performed appropriately and rigorously?

Reviewer #2: Yes

Author’s response: We want to thank the reviewer.

4. Have the authors made all data underlying the findings in their manuscript fully available?

The [PLOS Data policy](http://www.plosone.org/static/policies.action#sharing) requires authors to make all data underlying the findings described in their manuscript fully available without restriction, with rare exception (please refer to the Data Availability Statement in the manuscript PDF file). The data should be provided as part of the manuscript or its supporting information, or deposited to a public repository. For example, in addition to summary statistics, the data points behind means, medians and variance measures should be available. If there are restrictions on publicly sharing data—e.g. participant privacy or use of data from a third party—those must be specified.

Reviewer #2: Yes

Author’s response: We want to thank the reviewer.

5. Is the manuscript presented in an intelligible fashion and written in standard English?

PLOS ONE does not copyedit accepted manuscripts, so the language in submitted articles must be clear, correct, and unambiguous. Any typographical or grammatical errors should be corrected at revision, so please note any specific errors here.

Reviewer #2: Yes

Author’s response: We want to thank the reviewer.

6. Review Comments to the Author

Please use the space provided to explain your answers to the questions above. You may also include additional comments for the author, including concerns about dual publication, research ethics, or publication ethics. (Please upload your review as an attachment if it exceeds 20,000 characters)

Reviewer #2: (a) This paper is still very difficult to review and I don't agree with the journal requirements that the figures have to be not embedded in the text. It is a no brainer that it is far easier to read a paper with the figures in the right place. (b) I still see no point in publishing the received power levels but at least the total path loss is shown in the final column. (c) Also units dB and dBm need to be indicated in the headings of the tables or in the captions. The path loss exponents are eventually shown that are the important point of documentation in this work.

(d) Subscripts as words like "loss" and acronyms like "FSPL" in the equations should not be italic. This all needs to be consistent.

Author’s response: (a) We have nothing to do as the format set by the journal. (b) We have removed the path loss in Table 1, 2, and 3. (c) We have added power level measured in dB in Table 4, 5, and 6. (d) We have made the mentioned symbols, and other subscripts such as ABG, ABGM, CIDF, and CIDMF in equations (1)—(6), in the text below of these equations, and also in Table 8.

**Reviewer #3**

1. If the authors have adequately addressed your comments raised in a previous round of review and you feel that this manuscript is now acceptable for publication, you may indicate that here to bypass the “Comments to the Author” section, enter your conflict of interest statement in the “Confidential to Editor” section, and submit your "Accept" recommendation.

Reviewer #3: All comments have been addressed

Author’s response: We want to thank the reviewer.

2. Is the manuscript technically sound, and do the data support the conclusions?

The manuscript must describe a technically sound piece of scientific research with data that supports the conclusions. Experiments must have been conducted rigorously, with appropriate controls, replication, and sample sizes. The conclusions must be drawn appropriately based on the data presented.

Reviewer #3: Yes

Author’s response: We want to thank the reviewer.

3. Has the statistical analysis been performed appropriately and rigorously?

Reviewer #3: Yes

Author’s response: We want to thank the reviewer.

4. Have the authors made all data underlying the findings in their manuscript fully available?

The [PLOS Data policy](http://www.plosone.org/static/policies.action#sharing) requires authors to make all data underlying the findings described in their manuscript fully available without restriction, with rare exception (please refer to the Data Availability Statement in the manuscript PDF file). The data should be provided as part of the manuscript or its supporting information, or deposited to a public repository. For example, in addition to summary statistics, the data points behind means, medians and variance measures should be available. If there are restrictions on publicly sharing data—e.g. participant privacy or use of data from a third party—those must be specified.

Reviewer #3: No

Author’s response: We have already added the measured datasets in Table 1, 2, and 3.

5. Is the manuscript presented in an intelligible fashion and written in standard English?

PLOS ONE does not copyedit accepted manuscripts, so the language in submitted articles must be clear, correct, and unambiguous. Any typographical or grammatical errors should be corrected at revision, so please note any specific errors here.

Reviewer #3: Yes

Author’s response: We want to thank the reviewer.

6. Review Comments to the Author

Please use the space provided to explain your answers to the questions above. You may also include additional comments for the author, including concerns about dual publication, research ethics, or publication ethics. (Please upload your review as an attachment if it exceeds 20,000 characters)

Reviewer #3: The paper is revised well and improved by the authors which make it now qualified . So, I recommend it for publication

Author’s response: We want to thank the reviewer.
